# Use of Human Papillomavirus DNA, E6/E7 mRNA, and p16 Immunocytochemistry to Detect and Predict anal High-Grade Squamous Intraepithelial Lesions in HIV-Positive and HIV-Negative Men Who Have Sex with Men

**DOI:** 10.1371/journal.pone.0078291

**Published:** 2013-11-12

**Authors:** Nittaya Phanuphak, Nipat Teeratakulpisarn, Somboon Keelawat, Tippawan Pankam, Jiranuwat Barisri, Surang Triratanachat, Amornrat Deesua, Piyanee Rodbamrung, Jiratchaya Wongsabut, Patou Tantbirojn, Saranya Numto, Preecha Ruangvejvorachai, Praphan Phanuphak, Joel M. Palefsky, Jintanat Ananworanich, Stephen J. Kerr

**Affiliations:** 1 The Thai Red Cross AIDS Research Centre, Bangkok, Thailand; 2 SEARCH, Bangkok, Thailand; 3 Department of Pathology, Faculty of Medicine, Chulalongkorn University, Bangkok, Thailand; 4 Department of Obstetrics and Gynecology, Faculty of Medicine, Chulalongkorn University, Bangkok, Thailand; 5 HIV-NAT, Bangkok, Thailand; Department of Medicine, Faculty of Medicine, Chulalongkorn University, Bangkok, Thailand; 7 Department of Medicine, University of California San Francisco, San Francisco, California, USA; 8 The Kirby Institute for Infections and Immunity in Society, The University of New South Wales, Sydney, New South Wales, Australia; Shanghai Jiao Tong University School of Medicine, China

## Abstract

**Background:**

Men who have sex with men (MSM) are at high risk of having anal cancer. Anal high-grade squamous intraepithelial lesion (HSIL) is the precursor of anal cancer. We explored the use of different biomarkers associated with human papillomavirus (HPV) infection and HPV-mediated cell transformation to detect and predict HSIL among HIV-positive and HIV-negative MSM.

**Methodology/Principal Findings:**

A total of 123 HIV-positive and 123 HIV-negative MSM were enrolled and followed for 12 months. High-resolution anoscopy (HRA) with biopsies were performed at every visit along with anal sample collection for cytology, high-risk HPV DNA genotyping, HPV E6/E7 mRNA, and p16 immunocytochemistry. Performance characteristics and area under the receiver operator characteristics curve were calculated for these biomarkers at baseline, and Cox regression compared the usefulness of these biomarkers in predicting incident HSIL. High-risk HPV DNA, E6/E7 mRNA, and p16 immunocytochemistry each identified 43–46% of MSM whose baseline test positivity would trigger HRA referral. E6/E7 mRNA had the highest sensitivity (64.7%) and correctly classified the highest number of prevalent HSIL cases. With the exception of p16 immunochemistry, most tests showed significant increases in sensitivity but decreases specificity versus anal cytology, while the overall number of correctly classified cases was not significantly different. Baseline or persistent type 16 and/or 18 HPV DNA was the only test significantly predicting incident histologic HSIL within 12 months in models adjusted for HIV status and low-grade squamous intraepithelial lesions at baseline.

**Conclusions/Significance:**

Countries with a high HIV prevalence among MSM and limited HRA resources may consider using biomarkers to identify individuals at high risk of HSIL. E6/E7 mRNA had the highest sensitivity for prevalent HSIL detection regardless of HIV status, whereas type 16 and/or 18 HPV DNA performed best in predicting development of incident HSIL within 12 months.

## Introduction

Men who have sex with men (MSM) are at high risk of having anal cancer, and HIV-positive MSM have 5 times higher risk than HIV-negative MSM [1]. Anal high-grade squamous intraepithelial lesion (HSIL) is a putative precursor of anal cancer [2], [3], [4]. Both the prevalence and incidence of HSIL continue to increase in the era of highly active antiretroviral therapy (HAART) among MSM with HIV infection [5], [6], [7]. Persistent anal human papillomavirus (HPV) infection, especially with high-risk types, is the most important risk factor for HSIL and anal cancer [8], [9], [10], [11], [12], [13].

Anal cytology has generally been used first in the anal squamous intraepithelial lesion (SIL) screening algorithm [14], as it is relatively inexpensive and easy to perform. Abnormal cytology results trigger referral for high-resolution anoscopy (HRA) which permits visualization of abnormal tissue for biopsy. Once HSIL is diagnosed, treatment is generally provided in an attempt to prevent progression to anal cancer: limited data suggests a 9–15% progression from HSIL to anal cancer with a median follow-up of 3–5 years [2], [3], [4].

Similar to cervical cytology, low sensitivity and specificity for identifying those with biopsy-proven high-grade disease are commonly reported for anal cytology [15], [16]. The usefulness of other biomarkers for HSIL screening have therefore been evaluated but the data are limited [10], [15], [17], [18], [19], [20]. We prospectively studied the performance characteristics of high-risk HPV DNA testing, HPV E6/E7 oncogene mRNA testing, and p16 immunocytochemistry to identify individuals with prevalent HSIL in HIV-positive and HIV-negative MSM at an initial visit and to predict incident HSIL in this population over 12 months.

## Methods

### Ethics statement

All participants gave written informed consent. The study was approved by the institutional review board of Chulalongkorn University in Bangkok, Thailand (clinicaltrials.gov identification NCT01637298).

### Enrollment and follow-up of study participants

Thai men aged ≥18 years with a history of anal sex with men and documented HIV status were enrolled into the study at the Thai Red Cross AIDS Research Centre. MSM who had prior treatment for anal cancer, previous anal cytology or HRA were excluded from the study. Infrared coagulation ≤12 months before enrollment, intra-anal application of trichloroacetic acid or podophyllin ≤1 month before enrollment, or evidence of current intra-anal or perianal bacterial or herpes simplex virus anal infection at enrollment, were also exclusion criteria.

Participants were followed at month-12 after baseline except for the first 120 participants who were also scheduled for month-6 follow-up. Anal sample collection, HRA and HRA-guided biopsy of visible lesions were performed at all visits by the same study physician (NT).

### Anal sample collection and HRA

A moistened, non-lubricated swab (Rovers^®^ EndoCervex-Brush^®^, Rovers Medical Devices B.V., The Netherlands, or FLOQSwabs^TM^, Copan Italia S.p.A., Italy) was used to collect anal samples. Swabs were placed into liquid-based cytology fluid (Liqui-PREP^TM^, LGM International, Inc., Florida, USA) and stored at 4°C for ≤7 days before processing for anal cytology and p16 immunocytochemistry. Remaining cytology fluid was stored at −80°C until processing for HPV DNA and HPV E6/E7 mRNA. HRA was performed immediately after anal sample collection, using 5% acetic acid and Lugol's solution to aid visualization of abnormal anal tissue for biopsy.

### Diagnosis of anal low-grade squamous intraepithelial lesion (LSIL) and HSIL

Anal cytology results were classified using the 2001 Bethesda system [21] as normal, atypical squamous cells of undetermined significance (ASC-US), atypical squamous cells cannot exclude high-grade squamous intraepithelial lesion (ASC-H), low-grade squamous intraepithelial lesion (LSIL), high-grade squamous intraepithelial lesion (HSIL), or carcinoma. Histologic diagnoses were classified as normal, anal intraepithelial neoplasia (AIN) 1, AIN 2, or AIN 3. Diagnoses were given by three different pathologists and discrepancies resolved by re-evaluation, discussion, and concurrence by at least two pathologists.

A diagnosis of histologic HSIL was made based on a diagnosis of AIN 2 or AIN 3 on histology; histologic LSIL was AIN 1. The highest histologic grade reported was used for participants with >1 biopsy.

### HPV DNA genotyping

HPV genotyping was done using the LINEAR ARRAY^®^ HPV Genotyping Test (Roche Molecular Systems, Inc., Branchberg, NJ, USA), which amplifies target DNA within the polymorphic L1 region of the HPV genome, and subsequently hybridizes this product to probes for 37 anogenital HPV DNA genotypes (13 high-risk genotypes-16, 18, 31, 33, 35, 39, 45, 51, 52, 56, 58, 59, and 68–and 24 low-risk genotypes- HPV 6, 11, 26, 40, 42, 53, 54, 55, 61, 62, 64, 66, 67, 69, 70, 71, 72, 73 (MM9), 81, 82 (MM4), 83 (MM7), 84 (MM8), IS39 and CP6108). Primers for human β-globin gene were used for quality control.

### Quantification of intracellular HPV E6/E7 mRNA

An aliquot of liquid-based cytology fluid was used for intracellular HPV E6/E7 mRNA flow cytometry. Cell pellets were prepared, fixed and permeabilized. Fluorescence *in situ* hybridization for E6/E7 mRNA was performed using a cocktail of 5′- and 3′-labeled oligonucleotide probes (HPV OncoTect^TM^ E6, E7 mRNA Kit, IncellDx, Menlo Park, CA, USA). The kit covers the detection of E6/E7 mRNA from HPV types 16, 18, 31, 33, 35, 39, 45, 51, 52, 56, 58, 59, 68, and 69. Three-color flow cytometry was performed on Beckman Coulter Cytomics FC500. Cells were included in the analysis if they exhibited green fluorescence (fluorescein, HPV E6/E7 mRNA+) and blue fluorescence (4′-6-diamidino-2-phenylindole dihydrochloride, all cells) but lacked red fluorescence (polymorphonuclear cells). Samples with ≥2% of cells exhibiting E6/E7 mRNA were considered positive.

### p16 immunocytochemistry

Slides were prepared for p16 immunocytochemistry only from samples obtained at the baseline visit by the Immunohistochemistry and Immunocytochemistry Laboratory, Department of Pathology, Chulalongkorn University. The monoclonal antibody p16INK4a was used as a primary reagent and staining was performed using Bench Mark XT Instrument (Ventana, Medical System Inc., AZ, USA). p16 immunocytochemistry was scored by one anatomic pathologist (SK) unaware of the clinico-pathologic diagnosis and slides were considered p16-positive if cells with cytoplasmic and/or nuclear staining were present.

### Statistical Analysis

Statistical analysis was conducted with Stata version 12.1 (Statacorp, College Station, TX, USA). The prevalence and 12-month incidence of histologic HSIL in individuals without HSIL at baseline and 95% confidence intervals (95% CI) were calculated. The histologic diagnosis was used as the gold standard for comparing performance characteristics, and we assumed all participants with HSIL were identified.

Sensitivity, specificity, negative predictive value (NPV), positive predictive value (PPV), negative likelihood ratio (LR), and positive LR and 95% CI were calculated for the use of anal cytology, high-risk HPV DNA, intracellular HPV E6/E7 mRNA, and p16 immunocytochemistry to detect HSIL at baseline, for MSM overall and by HIV status. Receiver operator characteristics (ROC) plots were made to demonstrate sensitivity and specificity of these tests in detecting HSIL at baseline. Combinations of anal cytology, high-risk HPV DNA and E6/E7 mRNA to detect baseline and incident HSIL were also evaluated. Baseline differences in sensitivity and specificity between biomarkers were compared in subjects with HSIL and without HSIL respectively [22]; pairwise comparisons were made against anal cytology as a reference group. A formal comparison of the number of correctly classified responses, reflecting differences in both sensitivity and specificity, was made by comparing the area under the ROC curve (AROC). Cox Proportional Hazards regression with robust estimates of the variance was used to estimate the relative risk (Hazard ratio  =  HR) and 95% CI of incident HSIL in those with positive versus negative biomarker tests, for biomarkers individually and in combination. Models were made for baseline positive test results, and for persistent positive results before the development of HSIL.

## Results

### Participant characteristics

A total of 246 MSM (123 each HIV-positive and HIV-negative) were enrolled from December 2009– December 2010. Among the first 120 MSM (91 HIV-positive and 29 HIV-negative) scheduled for month-6 follow-up, 90 MSM (73 HIV-positive and 17 HIV-negative) attended the clinic. 167 MSM (89 HIV-positive and 78 HIV-negative) completed month-12 visit.

The mean (standard deviation, SD) age at enrollment was 28.8 (6.9) years for HIV-positive and 28.9 (7.4) years for HIV-negative MSM. The mean (SD) age of sexual debut was 18.0 (3.7) years for HIV-positive and 18.8 (3.7) years for HIV-negative MSM (p = 0.11). In HIV-positive MSM, the mean (SD) baseline CD4 count was 353 (146) cells/mm^3^ and 10% had baseline plasma HIV RNA <40 copies/mL. HAART use increased from 13% at baseline to 47% at month 12. Mean (SD) CD4 count at month 12 was 388 (130) cells/mm^3^ and 33% had plasma HIV RNA <40 copies/mL.

### Prevalence and incidence of histologic HSIL

HRA identified anal lesions in 55% (N = 136/246) of participants at baseline, 66% (N = 61/92) at month 6 and 56% (N = 94/167) at month 12 (Table 1). Abnormal HRA findings were more common in HIV-positive MSM versus HIV-negative MSM at each visit (67% vs. 43% at baseline, p<0.001, 72% vs. 50% at month 6, p = 0.08, and 68% vs. 39% at month 12, p<0.001).

**Table 1:** Anal cytology and histology results at baseline, month-6, and month-12 visits, by HIV status.

Baseline anal cyt ology				Baseline anal histology				
	Normal HRA, No Biopsy	Abnormal HRA, Refused Biopsy	Normal	AIN 1	AIN 2	AIN 3	Inadequate	*Total, n (%)*
***All MSM, n***								
Normal	104	1	21	55	13	13	0	*207 (84)*
ASC-US	1	0	0	12	1	4	0	*18 (7)*
LSIL	0	0	0	8	0	1	0	*9 (4)*
HSIL	0	0	0	3	0	0	0	*3 (1)*
Missing/Inadequate	5	0	1	1	1	1	0	*9 (4)*
*Total, n (%)*	*110 (45)*	*1 (0.4)*	*22 (9)*	*79 (32)*	*15 (6)*	*19 (8)*	*0 (0)*	*246 (100)*
***HIV-positive MSM, n***								
Normal	38	1	10	38	9	7	0	*103 (84)*
ASC-US	0	0	0	7	1	3	0	*11 (9)*
LSIL	0	0	0	1	0	1	0	*2 (2)*
HSIL	0	0	0	0	0	0	0	*0 (0)*
Missing/Inadequate	3	0	1	1	1	1	0	*7 (6)*
*Total, n (%)*	*41 (33)*	*1 (0.8)*	*11 (9)*	*47 (38)*	*11 (9)*	*12 (10)*	*0 (0)*	*123 (100)*
***HIV-negative MSM, n***								
Normal	66	0	11	17	4	6	0	*104 (85)*
ASC-US	1	0	0	5	0	1	0	*7 (6)*
LSIL	0	0	0	7	0	0	0	*7 (6)*
HSIL	0	0	0	3	0	0	0	*3 (2)*
Missing/Inadequate	2	0	0	0	0	0	0	*2 (2)*
*Total, n (%)*	*69 (56)*	*0 (0)*	*11 (9)*	*32 (26)*	*4 (3)*	*7 (6)*	*0 (0)*	*123 (100)*

Month 6 anal cytology				Month 6 anal histology				
	Normal HRA, No Biopsy	Abnormal HRA, Refused Biopsy	Normal	AIN 1	AIN 2	AIN 3	Inadequate	*Total, n (%)*
***All MSM, n***								
Normal	29	1	7	19	4	8	1	*69 (75)*
ASC-US	1	0	1	8	2	0	1	*13 (14)*
LSIL	0	0	0	3	0	0	0	*3 (3)*
HSIL	0	0	1	1	0	0	0	*2 (2)*
Missing/Inadequate	1	0	0	1	1	2	0	*5 (5)*
*Total, n (%)*	*31 (34)*	*1 (1)*	*9 (10)*	*32 (35)*	*7 (8)*	*10 (11)*	*2 (2)*	*92 (100)*
***HIV-positive MSM, n***								
Normal	20	1	6	13	4	8	1	*53 (72)*
ASC-US	1	0	1	6	2	0	1	*11 (15)*
LSIL	0	0	0	3	0	0	0	*3 (4)*
HSIL	0	0	1	1	0	0	0	*2 (3)*
Missing/Inadequate	1	0	0	1	1	2	0	*5 (7)*
*Total, n (%)*	*22 (30)*	*1 (1)*	*8 (11)*	*24 (32)*	*7 (10)*	*10 (14)*	*2 (3)*	*74 (100)*
***HIV-negative MSM, n***								
Normal	9	0	1	6	0	0	0	*16 (89)*
ASC-US	0	0	0	2	0	0	0	*2 (11)*
LSIL	0	0	0	0	0	0	0	*0 (0)*
HSIL	0	0	0	0	0	0	0	*0 (0)*
Missing/Inadequate	0	0	0	0	0	0	0	*0 (0)*
*Total, n (%)*	*9 (50)*	*0 (0)*	*1 (6)*	*8 (44)*	*0 (0)*	*0 (0)*	*0 (0)*	*18 (100)*

Month 12 anal cytology				Month 12 anal histology				
	Normal HRA, No Biopsy	Abnormal HRA, Refused Biopsy	Normal	AIN 1	AIN 2	AIN 3	Inadequate	*Total, n (%)*
***All MSM, n***								
Normal	64	2	15	20	2	5	0	*108 (65)*
ASC-US	7	2	1	13	5	6	0	*34 (20)*
LSIL	0	1	0	9	1	3	1	*15 (9)*
HSIL	0	1	0	2	1	0	0	*4 (2)*
Missing/Inadequate	2	0	2	2	0	0	0	*6 (4)*
*Total, n (%)*	*73 (44)*	*6 (4)*	*18 (11)*	*46 (28)*	*9 (5)*	*14 (8)*	*1 (0.6)*	*167 (100)*
***HIV-positive MSM, n***								
Normal	23	0	5	14	1	4	0	*47 (53)*
ASC-US	3	1	0	11	5	6	0	*26 (29)*
LSIL	0	1	0	8	0	3	1	*13 (15)*
HSIL	0	0	0	1	1	0	0	*2 (2)*
Missing/Inadequate	1	0	0	0	0	0	0	*1 (1)*
*Total, n (%)*	*27 (30)*	*2 (2)*	*5 (6)*	*34 (38)*	*7 (8)*	*13 (15)*	*1 (1)*	*89 (100)*
***HIV-negative MSM, n***								
Normal	41	2	10	6	1	1	0	*61 (78)*
ASC-US	4	1	1	2	0	0	0	*8 (10)*
LSIL	0	0	0	1	1	0	0	*2 (3)*
HSIL	0	1	0	1	0	0	0	*2 (3)*
Missing/Inadequate	1	0	2	2	0	0	0	*5 (6)*
*Total, n (%)*	*46 (59)*	*4 (5)*	*13 (17)*	*12 (15)*	*2 (3)*	*1 (1)*	*0 (0)*	*78 (100)*

Percentages are rounded and may not always add up to 100%.

AIN, anal intraepithelial neoplasia; HIV+, HIV-positive; HIV-, HIV-negative; ASC-US, atypical squamous cells of undetermined significance; LSIL, low-grade squamous intraepithelial lesion; HSIL, high-grade squamous intraepithelial lesion.

Baseline prevalence of HSIL was 18.9% in HIV-positive and 8.9% in HIV-negative MSM (p = 0.03). Over the study period, 28.8% of HIV-positive and 4.1% of HIV-negative MSM with no HSIL at baseline, developed HSIL.

### Baseline anal cytology, high-risk HPV DNA, E6/E7 mRNA, and p16 immunocytochemistry

#### Anal cytology

Abnormal anal cytology defined as ASC-US or worse was found in 12.7% (11.2% of HIV-positive and 14.1% of HIV-negative) MSM at baseline (Table 2). HIV-positive MSM without SIL or with LSIL had lower rates of abnormal anal cytology (10.7%) compared with those with HSIL (23.8%), p = 0.02.

**Table 2:** Baseline positivity of anal cytology and biomarkers by histologic anal diagnosis.

Anal diagnosis	No SIL	LSIL	HSIL	Total	P
*All MSM, n/N (%)*					
Anal cytology	1/127 (0.8)	23/78 (29.5)	6/32 (18.8)	30/237 (12.7)	0.27
High-risk HPV DNA	52/133 (39.1)	45/79 (57)	17/34 (50)	114/245 (46.3)	0.65
HPV 16 and/or 18 DNA	18/132 (13.6)	23/77 (29.8)	8/34 (23.5)	49/243 (20.3)	0.59
E6/E7 mRNA	47/132 (35.6)	42/78 (53.8)	22/34 (64.7)	111/244 (45.5)	0.02
p16 immunocytochemistry	42/103 (40.8)	34/64 (53.1)	9/29 (31)	85/196 (43.4)	0.15
High-risk HPV DNA and/or E6/E7 mRNA	78/132 (59.1)	64/79 (81)	28/34 (82.3)	174/245 (69.4)	0.08
Anal cytology and/or high-risk HPV DNA	52/133 (39.1)	55/79 (69.7)	21/34 (61.7)	128/246 (52.0)	0.22
Anal cytology and/or E6/E7 mRNA	48/133 (36.1)	51/79 (64.6)	25/34 (73.5)	124/246 (50.4)	0.004
*HIV-positive MSM, n/N (%)*					
Anal cytology	0/49 (0)	8/26 (30.7)	5/21 (23.8)	13/116 (11.2)	0.02.38
High-risk HPV DNA	26/53 (49.1)	30/47 (63.8)	13/23 (56.5)	69/123 (56.1)	0.96
HPV 16 and/or 18 DNA	11/52 (21.1)	15/45 (33.3)	7/23 (30.4)	33/120 (27.5)	0.73
E6/E7 mRNA	19/53 (35.8)	25/46 (54.4)	16/23 (69.6)	60/122 (49.2)	0.03
p16 immunocytochemistry	17/42 (40.5)	19/42 (45.2)	8/21 (38.1)	44/105 (41.9)	0.69
High-risk HPV DNA and/or E6/E7 mRNA	36/53 (67.9)	41/47 (87.2)	20/23 (86.9)	97/123 (78.9)	0.40
Anal cytology and/or high-risk HPV DNA	26/53 (49.1)	33/47 (70.2)	16/23 (69.5)	75/123 (60.9)	0.35
Anal cytology and/or E6/E7 mRNA	19/53 (35.8)	29/47 (61.7)	19/23 (82.6)	67/123 (54.4)	0.003
*HIV-negative MSM, n/N (%)*					
Anal cytology	1/78 (1.3)	15/32 (46.9)	1/11 (9.1)	17/121 (14.1)	1.00
High-risk HPV DNA	26/80 (32.5)	15/32 (46.9)	4/11 (36.4)	45/123 (36.5)	1.00
HPV 16 and/or 18 DNA	7/80 (8.8)	8/32 (25)	1/11 (9.1)	16/123 (13)	1.00
E6/E7 mRNA	28/79 (35.4)	17/32 (53)	6/11 (54.5)	51/122 (41.8)	0.52
p16 immunocytochemistry	25/61 (41)	15/22 (68.2)	1/8 (12.5)	41/91 (45)	0.07
High-risk HPV DNA and/or E6/E7 mRNA	42/79 (53.1)	23/32 (71.9)	8/11 (72.7)	73/122 (59.8)	0.52
Anal cytology and/or high-risk HPV DNA	26/80 (32.5)	22/32 (68.7)	5/11 (45.5)	53/123 (43.1)	1.00
Anal cytology and/or E6/E7 mRNA	29/80 (36.2)	22/32 (68.7)	6/11 (54.5)	57/123 (46.3)	0.75

P values correspond to a comparison of the proportion of subjects with no SIL or LSIL versus those with HSIL, who were identified by each biomarker.

SIL, squamous intraepithelial lesion; LSIL, low-grade squamous intraepithelial lesion; HSIL, high-grade squamous intraepithelial lesion; HPV, human papillomavirus.

LSIL group included MSM with anal intraepithelial neoplasia (AIN) 1 on histology. HSIL group included MSM with AIN 2 or AIN 3 on histology. No SIL group included MSM without LSIL or HSIL.

#### High-risk HPV DNA genotyping

High-risk HPV types were detected in 46.3% of MSM at baseline (56.1% of HIV-positive and 36.5% of HIV-negative MSM) (Table 2). HPV 16 and/or 18 infections were detected in 20.3% of MSM (27.5% of HIV-positive and 13% of HIV-negative MSM). High-risk HPV types were identified in 39.1% of MSM without SIL, 57% of MSM with LSIL and 50% of MSM with HSIL. HPV 16 and/or 18 infections were detected in 13.6% of MSM without SIL, 29.8% of MSM with LSIL and 23.5% of MSM with HSIL.

#### E6/E7 mRNA

Baseline E6/E7 mRNA was positive in 45.5% of MSM (49.2% of HIV-positive and 41.8% of HIV-negative MSM) (Table 2). E6/E7 mRNA positivity was higher in MSM with HSIL (64.7%) than those with LSIL (53.8%) or without SIL (35.6%), p = 0.02. The same trend was seen in HIV-positive MSM (p = 0.03).

#### p16 immunocytochemistry

Positive p16 immunocytochemistry was found among 43.4% of MSM at baseline (41.9% of HIV-positive and 45% of HIV-negative MSM) (Table 2). Positivity rates of p16 immunocytochemistry were not different between MSM with and without HSIL.

### Performance characteristics of individual biomarkers to detect baseline histologic HSIL

**Figure 1:**
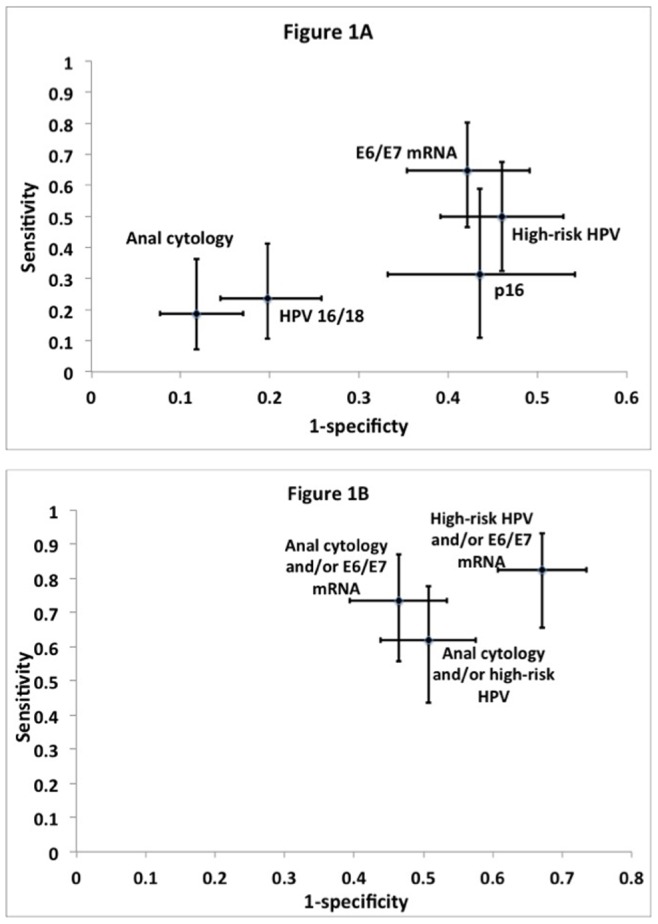
Receiver operator characteristics plots of biomarker performances to detect anal histologic HSIL. (A) Performance characteristics of anal cytology, high-risk HPV DNA, HPV 16 and/or 18, E6/E7 mRNA, and p16 immunocytochemistry, when each test was used alone to detect HSIL. (B) Performance characteristics when tests were used in combination to detect HSIL. HSIL, high-grade squamous intraepithelial lesion. HSIL included anal intraepithelial neoplasia (AIN) 2 or AIN 3 on histology.

Among all MSM at baseline, anal cytology had a sensitivity of 18.8% and a specificity of 88.2% to detect HSIL. E6/E7 mRNA was the most sensitive test (64.7%), followed by high-risk HPV DNA (50%), p16 immunocytochemistry (31.3%), and HPV 16 and/or 18 (23.5%) (Table 3). Specificity was approximately the same for E6/E7 mRNA (57.9%), high-risk HPV DNA (54%), and p16 immunocytochemistry (56.5%). AROC was highest for E6/E7 mRNA, indicating the highest proportion of correctly classified cases, but this was not significantly better than anal cytology (Table 3). Figure 1A demonstrates the performance characteristics of each test in detecting baseline HSIL. The overall PPVs of these tests were low and ranged from 11.1% to 20%. The PPVs tended to be higher in HIV-positive (ranged from 18.2% to 38.5%) than HIV-negative MSM (ranged from 2.4% to 11.8%). p16 immunocytochemistry had the lowest PPV while E6/E7 mRNA had the highest PPV, compared with the other tests. In all MSM and HIV-positive MSM, significant increases in sensitivity versus anal cytology were observed with all biomarkers and combinations, except HPV 16 and/or 18 and p16 immunocytochemistry. Significant decreases in specificity were observed for all biomarkers relative to anal cytology, so the AROC for biomarkers and combinations overall was not significantly different except for p16 immunocytochemistry which performed worse than anal cytology. In the HIV-negative MSM, sensitivity of biomarkers relative to anal cytology was not significantly different, but significant decreases in specificity relative to anal cytology were noted in all biomarkers except for HPV 16 and/or 18. As in the case of all MSM, no differences in the proportion of correctly classified responses were noted, except with p16 immunocytochemistry.

**Table 3:** Performance characteristics of anal cytology, high-risk HPV DNA, HPV E6/E7 mRNA, and p16 immunocytochemistry to detect anal histologic HSIL at baseline, by HIV status.

Test	Sensitivity^d^	Specificity^d^	PPV	NPV	Likelihood Ratios		AROC	P^e^
	% (95% CI)	% (95% CI)	% (95% CI)	% (95% CI)	Positive	Negative	(95% CI)	
*All MSM*								
Anal cytology	18.8 (7.2–36.4)	88.2 (83–92.3)	20 (7.7–38.6)	87.4 (82.1–91.6)	1.59	0.92	0.53 (0.46–0.60)	Ref
High-risk HPV DNA^a^	50 (32.4–67.6)*	54 (47.1–60.9)‡	14.9 (8.9–22.8)	87 (80–92.3)	1.09	0.93	0.52 (0.43–0.61)	0.55
HPV 16 and/or 18 DNA	23.5 (10.7–41.2)	80.3 (74.2–85.5)*	16.3 (7.3–29.7)	86.5 (80.9–91)	1.19	0.95	0.52 (0.44–0.59)	0.63
E6/E7 mRNA^b^	64.7 (46.5–80.3)‡	57.9 (50.9–64.7)‡	20 (13–28.7)	91 (84.8–95.3)	1.54	0.61	0.61 (0.52–0.70)	0.31
p16 immunocytochemistry^c^	31.3 (11–58.7)	56.5 (45.8–66.8)‡	11.1 (3.71–24.1)	82.5 (70.9–90.9)	0.71	1.22	0.43 (0.33–0.52)	0.01
High-risk HPV DNA and/or E6/E7 mRNA	82.4 (65.5–93.2)‡	32.9 (26.5–39.7)‡	16.6 (11.3–23)	92 (83.4–97)	1.23	0.54	0.54 (0.46–0.62)	0.58
Anal cytology and/or high-risk HPV DNA	61.8 (43.6–77.8)‡	49.3 (42.4–56.2)‡	16.4 (10.5–24)	88.9 (81.7–93.9)	1.22	0.78	0.56 (0.47–0.64)	0.96
Anal cytology and/or E6/E7 mRNA	73.5 (55.6–87.1)‡	53.6 (46.6–60.4)‡	20.3 (13.6–28.5)	92.6 (86.5–96.6)	1.58	0.49	0.64 (0.55–0.71)	0.07
*HIV-positive MSM*								
Anal cytology	23.8 (8.22–47.2)	91.5 (83.9–96.3)	38.5 (13.9–68.4)	84.3 (75.8–90.8)	2.8	0.83	0.58 (0.48–0.67)	Ref
High-risk HPV DNA^a^	56.5 (34.5–76.8)	43.4 (33.5–53.8)‡	18.8 (10.4–30.1)	81.1 (68–90.6)	1.0	1.0	0.50 (0.39–0.61)	0.2
HPV 16 and/or 18 DNA	30.4 (13.2–52.9)	72.9 (62.9–81.5)‡	21.2 (8.98–38.9)	81.4 (71.6–89)	1.12	0.95	0.52 (0.41–0.62)	0.34
E6/E7 mRNA^b^	69.6 (47.1–86.8)‡	56.1 (45.7–66.1)‡	27.1 (16.4–40.3)	88.7 (78.1–95.3)	1.59	0.54	0.63 (0.52–0.73)	0.72
p16 immunocytochemistry^c^	38.1 (18.1–61.6)	57.1 (45.9–67.9)‡	18.2 (8.19–32.7)	78.7 (66.3–88.1)	0.89	1.08	0.48 (0.36–0.59)	0.13
High-risk HPV DNA and/or E6/E7 mRNA	87 (66.4–97.2)‡	23.2 (15.3–32.8)‡	20.8 (13.2–30.3)	88.5 (69.8–97.6)	1.13	0.56	0.55 (0.47–0.63)	0.55
Anal cytology and/or high-risk HPV DNA	69.6 (47.1–86.8)‡	40.4 (30.7–50.7)‡	21.3 (12.7–32.3)	85.1 (71.7–93.8)	1.17	0.75	0.55 (0.44–0.66)	0.4
Anal cytology and/or E6/E7 mRNA	82.6 (61.2–95)‡	52.5 (42.2–62.7)‡	28.8 (18.3–41.3)	92.9 (82.7–98)	1.74	0.33	0.68 (0.58–0.76)	0.17
*HIV-negative MSM*								
Anal cytology	9.1 (0.23–41.3)	85.5 (77.5–91.5)	5.9 (0.2–28.7)	90.4 (83–95.3)	0.63	1.06	0.47 (0.38–0.56)	Ref
High-risk HPV DNA^a^	36.4 (10.9–69.2)	63.4 (53.8–72.3)‡	8.9 (2.5–21.2)	91 (82.4–96.3)	0.99	1	0.49 (0.34–0.65)	0.82
HPV 16 and/or 18 DNA	9.1 (.23–41.3)	86.6 (78.9–92.3)	6.3 (0.2–30.2)	90.7 (83.5–95.4)	0.69	1.05	0.48 (0.38–0.57)	0.95
E6/E7 mRNA^b^	54.5 (23.4–83.3)	59.5 (49.7–68.7)‡	11.8 (4.4–23.9)	93 (84.3–97.7)	1.35	0.76	0.57 (0.41–0.73)	0.26
p16 immunocytochemistry^c^	12.5 (0.3–52.7)	51.8 (40.6–62.9)‡	2.4 (0.1–12)	86 (73.3–94.2)	0.23	1.69	0.32 (0.19–0.45)	0.05
High-risk HPV DNA and/or E6/E7 mRNA	72.7 (39–94)	41.1 (20.2–38.2)‡	11 (4.85–20.5)	93.9 (83.1–98.7)	1.24	0.66	0.57 (0.42–0.71)	0.25
Anal cytology and/or high-risk HPV DNA	45.5 (16.7–76.6)	57.1 (47.4–66.5)‡	9.4 (3.13–20.7)	91.4 (82.3–96.8)	1.06	0.96	0.51 (0.35–0.67)	0.65
Anal cytology and/or E6/E7 mRNA	54.5 (23.4–83.3)	54.5 (44.8–63.9)‡	10.5 (3.96–21.5)	92.4 (83.2–97.5)	1.2	0.84	0.55 (0.38–0.70)	0.4

HSIL, high-grade squamous intraepithelial lesion; CI, confidence interval; PPV, positive predictive value; NPV, negative predictive value; AROC, area under the receiver operator characteristics curve; HPV, human papillomavirus.

HSIL included anal intraepithelial neoplasia (AIN) 2 or AIN 3 on histology.

a High-risk HPV DNA included HPV types 16, 18, 31, 33, 35, 39, 45, 51, 52, 56, 58, 59, and 68.

b E6/E7 mRNA positivity was defined as greater than or equal to 2% of cells which had E6/E7 mRNA over-expression in the sample.

c p16 immunocytochemistry was considered positive if there was a presence of cells with cytoplasmic and/or nuclear staining.

d *P≤0.05 and ‡ P≤0.001 in pairwise comparisons against anal cytology, in subjects with HSIL for sensitivity and subjects without HSIL for specificity.

e P values for pairwise comparisons of the AROC relative to anal cytology as a reference group.

### Biomarkers to predict incident histologic HSIL

We developed proportional hazards models for the usefulness of baseline positive biomarkers, and persistent positive biomarkers test results at two consecutive visits, in predicting progression to HSIL within 12 months. Based on our previous work where the only subject demographic, disease related and behavioral characteristics associated with development of HSIL were HIV-status and LSIL at baseline [23], multivariate models were developed adjusting for these covariates.

Among MSM without HSIL at baseline, a baseline positive high-risk HPV DNA or positive type 16 and/or 18 HPV DNA were the only biomarkers associated with an increased risk of developing HSIL in the following year in univariate models. When adjusted for HIV status and baseline LSIL, only baseline positive type 16 and/or 18 HPV DNA was associated with an increased risk of developing HSIL within 12 months (HR 2.48, 95% CI 1.04–5.96, p = 0.04) (Table 4).

**Table 4:** Baseline positive and persistent positive biomarkers predicting incident anal histologic HSIL at subsequent visits among those who were free of disease at baseline.

Biomarkers	Univariate		Multivariate^d^	
	HR (95% CI)	P	HR (95% CI)	P
**Baseline biomarkers**				
Anal cytology	1.48 (0.52–4.19)	0.46	1.24 (0.39–3.91)	0.71
High-risk HPV DNA^a^	3.22 (1.28–8.11)	0.01	2.00 (0.76–5.26)	0.158
HPV 16 and/or 18 DNA	3.59 (1.57–8.19)	0.002	2.48 (1.04–5.96)	0.04
E6/E7 mRNA^b^	1.02 (0.44–2.35)	0.96	0.98 (0.43–2.24)	0.97
p16 immunocytochemistry^c^	0.87 (0.36–2.08)	0.75	0.82 (0.35–1.93)	0.648
High-risk HPV DNA and/or E6/E7 mRNA	1.86 (0.66–5.25)	0.24	1.16 (0.39–3.46)	0.789
Anal cytology and/or high-risk HPV DNA	2.22 (0.93–5.32)	0.07	1.37 (0.55–3.41)	0.49
Anal cytology and/or E6/E7 mRNA	1.22 (0.53–2.82)	0.65	1.04 (0.45–2.37)	0.93
**Persistent positive biomarkers**				
Anal cytology	3.95 (1.64–9.51)	0.002	2.54 (0.86–7.51)	0.09
High-risk HPV DNA^a^	3.85 (1.56–9.47)	0.003	2.41 (0.96–6.04)	0.06
HPV 16 and/or 18 DNA	6.44 (2.77–14.96)	<0.001	4.48 (1.90–10.59)	0.001
E6/E7 mRNA^b^	1.19 (0.53–2.67)	0.68	1.02 (0.44–2.36)	0.96
High-risk HPV DNA and/or E6/E7 mRNA	2.94 (0.91–9.52)	0.07	1.55 (0.44–2.36)	0.49
Anal cytology and/or high-risk HPV DNA	4.12 (1.59–10.66)	0.003	2.25 (0.78–6.49)	0.14
Anal cytology and/or E6/E7 mRNA	1.37 (0.60–3.14)	0.46	0.95 (0.40–2.26)	0.91

HSIL, high-grade squamous intraepithelial lesion; HPV, human papillomavirus; CI, confidence interval.

HSIL included anal intraepithelial neoplasia (AIN) 2 or AIN 3 on histology.

a High-risk HPV DNA included HPV types 16, 18, 31, 33, 35, 39, 45, 51, 52, 56, 58, 59, and 68.

b E6/E7 mRNA positivity was defined as greater than or equal to 2% of cells which had E6/E7 mRNA over-expression in the sample.

c p16 immunocytochemistry was considered positive if there was a presence of cells with cytoplasmic and/or nuclear staining.

d Multivariate model adjusted for HIV status and low-grade squamous intraepithelial lesion at baseline.

Persistent positive abnormal anal cytology, high-risk HPV DNA, type 16 and/or 18 HPV DNA, and a combination of persistent abnormal anal cytology and/or high-risk HPV DNA were predicted progression to HSIL in univariate models. In adjusted models, only persistent type 16 and/or 18 HPV DNA predicted development of HSIL within 12 months (HR 4.48, 95% CI 1.90–10.59, p = 0.001) (Table 4).

## Discussion

The high prevalence (18.9%) and 12-month incidence (28.8%) of histologic HSIL observed among HIV-positive MSM in our study were within the range reported in Western studies [5], [7], [9], [24], [25]. Together, these data demonstrate the critical importance of implementing HSIL screening programs where HIV is common among MSM. In both resource-limited and resource-rich countries, there is a paucity of HRA equipment and clinicians trained in HRA. Therefore, biomarkers could potentially identify those with the highest risk of HSIL, who would benefit most from HRA referral.

Performance characteristics of biomarkers associated with anal HPV infection and HPV-mediated cell transformation demonstrated that high-risk HPV DNA, E6/E7 mRNA, and p16 immunocytochemistry, when used as a primary screening test to trigger referral of MSM at high risk for HSIL to HRA, identified comparable proportions (ranging from 43–46%) of men with baseline positivity. E6/E7 mRNA had higher sensitivity, higher PPV, and higher AROC than the other tests, and identified a significantly higher proportion of subjects with HSIL versus LSIL or no SIL than anal cytology (p = 0.02). Anal cytology only identified 12.7% of MSM for HRA referral with a lower sensitivity and lower AROC than E6/E7 mRNA, although with comparable PPV. Use of E6/E7 mRNA in combination with anal cytology provided a higher sensitivity and a higher proportion of correctly classified cases than when either test was used alone, and showed a significant difference in the proportion of subjects with HSIL versus LSIL or no SIL (p = 0.004).

Recent studies have compared the performances of E6/E7 mRNA and high-risk HPV DNA to detect HSIL among HIV-positive MSM. A US study demonstrated that E6/E7 mRNA provided a lower sensitivity than high-risk HPV DNA (79.8% vs. 100%) but with a higher PPV (50.9% vs. 40.3%) to detect HSIL, and a higher proportion of correctly classified cases [26]. Baseline E6/E7 mRNA positivity was 49.3%, and 79.1% for high-risk HPV DNA. In a German study, E6/E7 mRNA demonstrated similar sensitivity to high-risk HPV DNA but with a two-fold higher specificity (46.0% vs. 26.1%) and a higher PPV (25.2% vs. 19.7%) [19]. E6/E7 mRNA was detected in 60.6% and high-risk HPV DNA was detected in 77.3% of MSM in this study. Although direct comparisons with HIV-positive MSM in our study are limited by an approximate 20-year age gap and different laboratory techniques, the data agree insofar as none of these tests performed well to detect HSIL. E6/E7 mRNA, however, had a higher PPV and correctly classified more cases than high-risk HPV DNA, and resulted in fewer HRA referrals.

Among those with no HSIL at baseline, baseline or persistent type 16 and/or 18 HPV DNA was the only biomarker which predicted development of HSIL within 12 months. E6/E7 mRNA and p16 immunocytochemistry, which are considered biomarkers that identify transforming HPV infections and precancer, did not show a significant association. Types of high-risk HPV covered by these tests are unlikely to explain this finding as the high-risk HPV types covered by the HPV DNA genotyping test and the E6/E7 mRNA kit used in our study are almost identical and p16 immunocytochemistry is not type-restricted. Nevertheless, because of our small number of incident cases these data should be interpreted with caution.

HRA performance on all study participants and rigorous anal sampling for different biomarkers by the same study physician is a major strength of our study. Nevertheless, our study has several limitations. First, while a recent study demonstrated that random biopsies during HRA significantly increased the rate of HSIL detection, only 55% of participants who underwent HRA at baseline in our study had abnormal findings which were biopsied [27]. A lower biopsy rate compared with previous studies might indicate that some cases, later included as incident cases were missed at baseline [5], [26]. With just over 1-year experience in HRA of our study physician, it was likely that there was a learning curve as the study went along as increasing proportions of participants with HSIL cytology who had HSIL histology were observed over the study period. Such a scenario resulting in performance changes of HRA over the study period may affect the overall performance characteristics of the studied biomarkers to detect prevalent and incident HSIL. Similarly, the anal cytology positivity rate increased over time in this cohort, which might reflect improvements in the ability of our physician to collect adequate cells for cytology. This might impair the true sensitivity of anal cytology and explain the overall lower sensitivity of anal cytology seen in our study compared with previous studies [14], [15]. Second, although p16 immunohistochemistry can enhance the accuracy of histologic diagnoses of both cervical and anal biopsy specimens [28], [29], we did not have the opportunity to incorporate this into the histologic diagnoses used as the gold standard in our study. Third, the 68% follow-up rate of participants over a 12-month period in our study might bias our results describing the relative risk of developing HSIL over the study period. However, we did not see any difference in baseline characteristics between those who lost to follow-up and those who were retained in the study (data not shown). In addition, our inclusion of month-6 data from a subset of participants might also result in a bias of the overall predictive power of persistent biomarkers to detect incident HSIL. Nonetheless, long-term follow-up of this cohort will further strengthen the understanding of biomarkers used to detect HSIL among HIV-positive and HIV-negative MSM. Fourth, the generalizability of the HPV DNA test used in this study may be limited since we used the LINEAR ARRAY^®^ HPV Genotyping Test which is less readily available in most countries than the other HPV DNA tests such as Hybrid Capture^®^ 2. The LINEAR ARRAY^®^ HPV Genotyping Test was selected for its ability to identify each of the 37 HPV types, as one of our study cohort's objectives was to characterize HPV types distribution in the anal canal of Thai MSM. Lastly, the data presented in this study may not be applicable to other settings with different anal HPV infection and HSIL epidemics than described here.

In summary, we demonstrated that the use of E6/E7 mRNA seemed to correlate best with prevalent histologic HSIL detection both among HIV-positive and HIV-negative MSM, and a significantly higher proportion of subjects with HSIL than LSIL or no SIL showed baseline E6/E7 mRNA. For predicting development of incident histologic HSIL at 12-months, baseline and persistent type 16 and/or 18 HPV DNA were significantly better than other biomarkers studied. Countries with high prevalence of HIV infection among MSM and limited resources for HRA may benefit from using biomarkers in their HSIL screening programs for MSM. As none of the biomarkers evaluated in our study performed at high level, additional studies are in need to identify new biomarkers or combinations of biomarkers with better performances to correctly trigger referral for HRA.
